# Generation, Characterization, and Preclinical Studies of a Novel NKG2A-Targeted Antibody BRY805 for Cancer Immunotherapy

**DOI:** 10.3390/antib13040093

**Published:** 2024-11-20

**Authors:** Yaqiong Zhou, Yiru Wang, Jinfeng Liang, Jing Qian, Zhenhua Wu, Zhangzhao Gao, Jian Qi, Shanshan Zhu, Na Li, Yao Chen, Gang Chen, Lei Nie, Tingting Guo, Haibin Wang

**Affiliations:** 1 BioRay Pharmaceutical Co., Ltd., Taizhou 318000, China; 2 BioRay Pharmaceutical (Hangzhou) Co., Ltd., Hangzhou 311404, China; 3 Faculty of Chinese Medicine, Macau University of Science and Technology, Macau, China; 4 Zhejiang Center for Drug and Cosmetic Evaluation, Zhejiang Medical Products Administration, Hangzhou 310012, China; 5 BioRay Pharmaceutical Corp., San Diego, CA 92121, USA

**Keywords:** cancer immunotherapy, checkpoint inhibitor, NKG2A, NK cells, HLA-E

## Abstract

Immuno-oncology has revolutionized cancer treatment, with NKG2A emerging as a novel target for immunotherapy. The blockade of NKG2A using the immune checkpoint inhibitor (ICI) monalizumab has been shown to enhance the responses of both NK cells and CD8+ T cells. However, monalizumab has demonstrated limited efficacy in in vitro cytotoxic assays and clinical trials. In our study, we discovered and characterized a novel anti-NKG2A antibody, BRY805, which exhibits high specificity for the human CD94/NKG2A heterodimer complex and does not bind to the activating NKG2C receptor. In vitro cytotoxicity assays demonstrated that BRY805 effectively activated NK92 cells and primary NK cells, thereby enhancing the cytotoxic activity of effector cells against cancer cells overexpressing HLA-E, with significantly greater efficacy compared to monalizumab. Furthermore, BRY805 exhibited synergistic antitumor activity when combined with PD-L1 monoclonal antibodies. In a mouse xenograft model, BRY805 showed superior tumor control relative to monalizumab and demonstrated a favorable safety profile in non-human primate studies.

## 1. Introduction

The primary mechanisms by which tumors evade immune surveillance encompass the following: the evasion of tumor cell recognition by the host immune system; the immunoregulatory effects exerted by tumor cells on leukocyte functions; the involvement of myeloid cells in tumor progression; the induction of apoptosis in immunocompetent cells; the evasion of apoptosis by tumor cells; and the modulation of immune checkpoints [1]. Among these mechanisms, the application of checkpoint inhibitors has constituted a significant advancement in cancer therapy over the past decade. Immunoinhibitors function by obstructing proteins that suppress immune responses. Frequently targeted proteins include PD-1 (programmed cell death protein 1), PD-L1 (programmed cell death ligand 1), and CTLA-4 (cytotoxic T-lymphocyte antigen-4). By inhibiting these checkpoints, immunoinhibitors enhance T-cell activation and proliferation, thereby enabling the immune system to more effectively recognize and eliminate cancer cells. Checkpoint inhibitors have markedly enhanced survival outcomes for patients with metastatic cancer. These include antibodies targeting the PD-1 /PD-L1 axis and CTLA-4, utilized either as monotherapy or in combination therapies approved for various cancer indications [2]. These treatments frequently result in durable benefits, and in the majority of patients, associated toxicities can be effectively managed. The effectiveness of immunoinhibitors can be influenced by biomarkers such as tumor mutational burden (TMB) and PD-L1 expression levels, helping to identify which patients are more likely to benefit from these therapies [3]. However, only a subset of patients exhibit a robust response and derive benefit from approved immune checkpoint inhibitors (ICIs) [4]. For the existence of various mechanisms of tumor escape, including downregulation of classical HLA class I molecules (required to present peptides derived from tumor-specific antigens to CTL) and the release of inhibitory cytokines [e.g., transforming growth factor (TGF)-b and/or soluble factors (e.g., prostaglandin E_2_ and kynurenine)] from tumor cells or cells of the tumor microenvironment (e.g., M2 macrophages, myeloid-derived suppressor cells). Another explanation may be a lack or low expression of ICI ligands on tumor cells (e.g., PD-L1) [5]. Consequently, numerous studies are underway to identify novel ICIs and develop combination strategies to enhance the efficacy of existing ICIs. Furthermore, all currently approved ICIs for cancer treatment primarily target the activation of T cells. In this context, the antitumor potential of natural killer (NK) cells remains underexplored. Recent research has identified NKG2A as a significant “checkpoint” on NK cells, demonstrating promising clinical outcomes with limited toxicity in highly aggressive human tumors.

NKG2A represents a novel immune checkpoint target with significant potential in the field of cancer immunotherapy. This inhibitory receptor is expressed on certain subsets of cytotoxic lymphocytes, notably natural killer (NK) cells and CD8+ T cells, and interacts with the non-classical major histocompatibility complex (MHC) class I molecule, HLA-E. This interaction is pivotal in modulating immune responses. By negatively regulating the activity of NK cells and T cells, NKG2A plays a critical role in both the evasion of tumors from immune detection and the surveillance of tumors by the immune system [6]. Cancer cells can evade immune surveillance by expressing ligands for NKG2A, thereby inhibiting the activation and cytotoxic functions of natural killer (NK) cells and CD8+ T cells, which permits unchecked tumor growth. Numerous carcinoma types are notably linked to poorer prognoses and diminished responses to immunotherapy. In particular, colorectal, breast, and clear cell renal cancers exhibit a negative prognostic association with HLA-E expression, which appears to facilitate tumor immune evasion. Targeting NKG2A may mitigate this evasion mechanism, thereby enhancing the antitumor effects mediated by NK and CD8+ T cells [7,8,9]. Preliminary clinical trials involving NKG2A antagonists have demonstrated potential in enhancing antitumor responses, with certain patients exhibiting sustained responses. These findings indicate that targeting NKG2A may constitute an effective strategy in cancer therapy. Furthermore, NKG2A blockade can be integrated with other immunotherapeutic approaches, such as PD-1/PD-L1 inhibitors and CTLA-4 inhibitors. This combinatorial approach may result in synergistic effects, thereby augmenting overall treatment efficacy.

A humanized anti-NKG2A IgG antibody, monalizumab, has been developed, and numerous clinical trials are currently underway across various tumor indications. Monalizumab is administered either as a monotherapy or in combination with other therapeutic antibodies, such as durvalumab, which targets PD-L1, or cetuximab, which targets the epidermal growth factor receptor (EGFR) expressed by tumor cells [10,11]. The concurrent administration of anti-NKG2A and anti-PD-L1 blocking antibodies activates NK and CD8 T cells, thereby inhibiting tumor progression in stage II non-small cell lung cancer [12]. Nevertheless, the use of monalizumab is accompanied by several limitations. There is significant variability in therapeutic responses among patients, as not all tumors express the necessary ligands or have sufficient immune cell infiltration to benefit from the treatment. Furthermore, data on the long-term efficacy and safety of monalizumab remain limited, highlighting the need for more comprehensive clinical trials. Monalizumab exhibited reduced efficacy in enhancing NK cell-mediated cytotoxicity against target cells presenting HLA-G peptides on HLA-E compared to cells expressing HLA-E complexed with HLA-A, HLA-B, and HLA-C peptides. This differential effectiveness may contribute to tumor resistance mechanisms [13]. Additionally, monalizumab demonstrated limited cytotoxic effects in mitigating natural killer cell dysfunction in chronic lymphocytic leukemia (CLL), as supported by our findings [14]. Consequently, clinical trials investigating monalizumab for CLL and other malignancies were discontinued, likely due to its insufficient therapeutic efficacy (NCT02557516, NCT02671435, NCT04590963). 

In pursuit of a more potent NKG2A inhibitor, we present the discovery and development of a novel anti-NKG2A antibody, BRY805, as a strategy to achieve therapeutic benefits through NKG2A:HLA-E blockade. BRY805 exhibits specific high-affinity binding to NKG2A, effectively inhibiting the interaction between NKG2A and HLA-E. In vitro assays demonstrate that the blockade of NKG2A by BRY805 can activate natural killer (NK) cells and augment NK cell-mediated antitumor effects in a dose-dependent manner. Furthermore, BRY805 significantly enhances the cytotoxicity of primary NK cells against tumor cells compared to monalizumab, both in vitro and in vivo. Additionally, BRY805 acts synergistically with PD-L1 monoclonal antibodies to potentiate antitumor cell activity. BRY805 is a novel and distinct NKG2A-targeting antibody that demonstrates promising efficacy and a favorable safety profile in both in vitro and in vivo studies. These findings support the continued development of NKG2A-based therapeutics for cancer treatment. BRY805 is currently being evaluated in a Phase 1 clinical study in China.

## 2. Materials and Methods

### 2.1. Cell Lines

Human NKG2A/CD94, Cynomolgus NKG2A/CD94 and human NKG2C/CD94 overexpressing CHO cells (CHOK1-hNKG2A/CD94, CHOK1-cynoNKG2A/CD94, CHOK1-hNKG2C/CD94), were generated by transducing CHO cells with full-length human or cynomolgus coding sequences. Human NKG2A overexpressing 293F cells (293F-hNKG2A/CD94) were generated by the transduction of 293F cells with full-length human NKG2A/CD94 coding sequences. LCL721.221 and MOLM-13 cells were obtained from ChemPartner (Shanghai, China). The overexpression of HLA-E in LCL721.221 and MOLM-13 cells was induced by HLA-A*02 or HLA-B*08 signal peptides. NCI-H1975 cells were obtained from Gempharmatech (Nanjing, China).

### 2.2. Antibodies

The amino acid sequences of monalizumab and avelumab (an anti-PD-L1 MAb) were sourced from the IMGT/mAb-DB database. The genes encoding the heavy and light chains were subsequently cloned into the mammalian expression vector pcDNA3.4. Expression was carried out using the ExpiCHO system (Invitrogen, Waltham, MA, USA), and the resulting proteins were purified via protein A chromatography (GE). These purified proteins were utilized as positive controls in this study. 

### 2.3. Antibody Development and Humanization

Anti-human NKG2A antibodies were produced using standard hybridoma technology. Three distinct immunogens were employed to immunize SJL mice: human NKG2A/CD94 DNA (ChemPartner, Shanghai, China); 293F-hNKG2A/CD94; and recombinant human NKG2A/CD94 extracellular domain protein (hNKG2A/CD94-ECD, ChemPartner). Hybridomas were subsequently selected, and the supernatants from the resulting clones were screened using NKG2A and NKG2C binding and blocking assays. One hybridoma clone, designated 349E1E3-1C4, was sequenced. The antibody was humanized through fusion with an IgG4 containing three single-point mutations in the Fc heavy chain and CDR-grafting into human germline frameworks. The resulting humanized antibodies were screened for binding affinity to NKG2A, aiming to achieve an affinity comparable to the original murine monoclonal antibody. The humanized antibody that met these criteria was designated BRY805. 

### 2.4. Generation of NKG2A-Specific Antibody

The coding sequences of BRY805 were subcloned into the pcDNA3.4 vector. Subsequently, the ExpiCHO expression system was employed to express BRY805 in accordance with the manufacturer’s protocol. Fourteen days post-transfection, the cell culture was harvested and subjected to purification using a Protein A affinity column. The purified product was then reconstituted in PBS buffer to facilitate long-term storage. 

### 2.5. Binding ELISA 

hNKG2A/CD94-ECD, Cynomolgus NKG2A/CD94-ECD (cynoNKG2A/CD94-ECD), or human NKG2E proteins (hNKG2E, sourced from ChemPartner), were diluted to a concentration of 1 μg/mL and subsequently coated onto a 96-well ELISA plate, where they were allowed to incubate overnight at 4 °C. Following incubation, the plate was subjected to a washing process and then blocked for 1 h using a solution of 5% bovine serum albumin in phosphate-buffered saline (BSA/PBS) at ambient temperature. Antibodies prepared in serial dilutions were introduced to the plate in duplicate and allowed to incubate for 1 h at room temperature. Subsequent to another washing step, Goat F(ab’)2 anti-human IgG Fc conjugated with horseradish peroxidase (HRP) (obtained from Abcam, Hangzhou, China) was added and incubated for an additional hour at room temperature. The plates were washed, and the substrate TMB solution was subsequently added, followed by an incubation period of 0.5 h. The reaction was then terminated, and its absorbance was measured at 450 nm using a Bio-Rad iMARK plate reader (Bio-Rad, Berkeley, CA, USA).

### 2.6. Cell-Binding Assay

CHOK1-hNKG2A/CD94, CHOK1-cynoNKG2A/CD94, CHOK1-hNKG2C/CD94, or CHOK1-blank cells were incubated for 30 min with a serial dilution of antibodies. Post-washing, the cells were further incubated with Goat anti-Human IgG Secondary Antibody, Alexa Fluor™ 488 (Invitrogen) for 30 min at 4 °C. The stained cells were analyzed using a CytoFlex system (Beckman, Brea, CA, USA). Median fluorescence intensity values were plotted against the concentration of the primary antibody.

### 2.7. Cell-Based Receptor Blocking Assay

CHOK1-hNKG2A/CD94 cells were rinsed and resuspended in FACS buffer(PBS supplemented with 2% FBS). The cells were then distributed into 96-well round-bottom plates and incubated with a diluted antibody solution at 4 °C for 30 min. Subsequently, HLA-E PE was added to the plates and incubated with the cells at 4 °C for an additional 2.5 h. Following incubation, the cells were washed and subjected to fluorescence-activated cell sorting (FACS) analysis.

### 2.8. NK-92 Cell-Mediated Cytotoxicity Assay of BRY805

HLA-E overexpressing LCL721.221 or MOLM-13 cells were labeled with a fluorescence-enhancing ligand (DELFIA BATDA Reagent, Revvity, Waltham, MA, USA) in assay buffer and incubated for 20 min at 37 °C in a cell incubator. Subsequently, the cells were washed with PBS and resuspended in RPMI 1640 supplemented with 10% FBS. Serial dilutions of BRY805 or control antibodies were introduced into the assay plate and incubated with NK92 cells at 37 °C for 30 min. Following this, LCL721.221 or MOLM-13 cells were added to the assay plate and incubated at 37 °C for a duration of 2 to 4 h. The cell supernatants were then transferred to a flat-bottom detection plate, to which Europium Solution was added. The plate was agitated at 250 rpm for 15 min at room temperature, and fluorescence was measured using a time-resolved fluorometer within 5 h.

### 2.9. Primary NK Cell-Mediated Cytotoxicity Assay of BRY805

HLA-E overexpressing LCL721.221 cells were labeled in assay buffer using the fluorescence-enhancing ligand (DELFIA BATDA Reagent) and incubated at 37 °C for 20 min in a cell incubator. Following this incubation, the cells were washed with PBS and subsequently resuspended in RPMI 1640 supplemented with 10% FBS. Serial dilutions of BRY805 or control antibodies were then introduced into the assay plate and incubated with NK92 cells at 37 °C for 30 min. After this incubation, LCL721.221 cells were added to the assay plate and incubated at 37 °C for an additional 2 to 4 h. Finally, the cell supernatants were transferred to a flat-bottom detection plate. Europium solution was then added to the detection plate, which was subsequently shaken at 250 rpm for 15 min at room temperature. Fluorescence measurements were taken using a time-resolved fluorometer within 5 h.

### 2.10. NK-92 Cell-Mediated Cytotoxicity Assay of BRY805 Combined with PD-L1 MAb 

Avelumab was employed as a PD-L1 monoclonal antibody (MAb) in the cytotoxicity assay. Initially, 50 μL of NK92 cells was introduced into a 96-well microplate at a concentration of 4E5 cells/mL. Subsequently, 250 ng of either BRY805 or control antibodies was added to the assay plate and incubated with the NK92 cells at 37 °C for 30 min. Following this, 100 μL of LCL721.221 cells, labeled with Calcein-AM (MedChemExpress, Monmouth, NJ, USA), was added to the assay plate at a concentration of 1E5 cells/mL. Finally, serially diluted avelumab or control antibodies were introduced into the assay plate and incubated at 37 °C for 70 min. The plate was centrifuged at 500× *g* for 3 min at room temperature, after which 100 μL of the culture supernatant was quantified using a Tecan microplate reader.

### 2.11. In Vivo Efficacy Studies

For the tumor xenograft model, the HLA-E expressing cell line NCI-H1975 was utilized to inoculate huHSC-NCG-hIL15 mice, which exhibited superior immune reconstitution levels of hNKG2A+ NK cells compared to other immune reconstitution mouse models. A total of 26 female huHSC-NCG-hIL15 mice (GemPharmatech Co., Ltd., Nanjing, China), aged 8–10 weeks and weighing approximately 25 g, were employed in this study. Each mouse was subcutaneously injected with 2 × 10^6^ NCI-H1975 cells in the right flank. On the twelfth day post-inoculation, when the mean tumor volume reached 57.57 mm^3^, a cohort of 21 mice was randomly assigned into three groups based on tumor volume, body weight, and the immune reconstitution level of NKG2A+ NK cells, with each group comprising seven mice (n = 7). The allocation methodology was provided by GemPharmatech Co., Ltd., and the remaining mice were euthanized. The day of group allocation was designated as day 0, with treatment commencing on the same day. Antibodies were administered intraperitoneally to the mice in the three groups, including the investigational drug BRY805, a positive control monalizumab analog, and a negative control hIgG4 isotype. The dosing volume for each group, as well as the sequence of treatments and measurements, remained consistent throughout this study. Treatments were administered biweekly over a three-week period, with the possibility of extending observation periods as necessary. The primary evaluation metrics for this experiment included tumor size and the overall health status of the test mice. Body weights of all models and tumor volumes were assessed twice weekly. At the conclusion of this experiment, tumor samples were collected and weighed.

Tumor volume (TV) measurements were obtained using calipers to ascertain the longest and shortest axes of the tumor, with the following formula employed for volume calculation: TV (mm^3^) = 0.5 × Length × Width^2^. The T_TV_/C_TV_ value is the ratio of the average tumor volume in the treatment group (TmTV) to the average tumor volume in the control group (CmTV), and the tumor growth inhibition value was defined as TGI_TV_%. T_TV_/C_TV_ = TmTV/CmTV; TGI_TV_% = (1 − T_TV_/C_TV_) × 100%. The T_TW_/C_TW_ value is the ratio of the average tumor weight in the treatment group (TmTW) to the average tumor weight in the control group (CmTW), and the tumor weight change value was defined as TGI_TW_%. T_TW_/C_TW_ = TmTW/CmTW, TGI_TW_% = (1 − T_TW_/C_TW_) × 100%

The experimental design, outcome assessment, and data analysis were assigned to a single individual, while the execution of this experiment was conducted by a different person. Experimental data, including tumor volume, body weight, and tumor weight of mice, are presented as mean ± SEM unless specified otherwise. Statistical analyses were conducted using GraphPad Prism 10 software, employing an unpaired two-sided Student’s *t*-test for evaluation.

### 2.12. Toxicity Study in Non-Human Primates

The non-human primate toxicity study was conducted using cynomolgus monkeys at Suzhou Xishan Zhongke Pharmaceutical Research and Development Co., Ltd. (Suzhou, China). In this study, groups of animals, consisting of five males and five females each, received a weekly intravenous infusion of BRY805 at a dosage of 50 mg/kg or a vehicle control composed of 10 mM L-Histidine at pH 6.0 over a period of five weeks, totaling five doses. After the dosing phase, two animals of each sex per group were retained for a subsequent 6-week recovery period. The in-life assessments encompassed clinical observations, measurements of body weight, food consumption analysis, cardiovascular safety pharmacology evaluations, ophthalmologic examinations, and clinical pathology, which included serum chemistry, hematology, coagulation, and urinalysis. Additionally, gross pathology, relative organ weight assessments, and histopathological examinations were conducted.

## 3. Results

### 3.1. Generation and Characterization of an NKG2A-Specific Antibody

**Figure 1:**
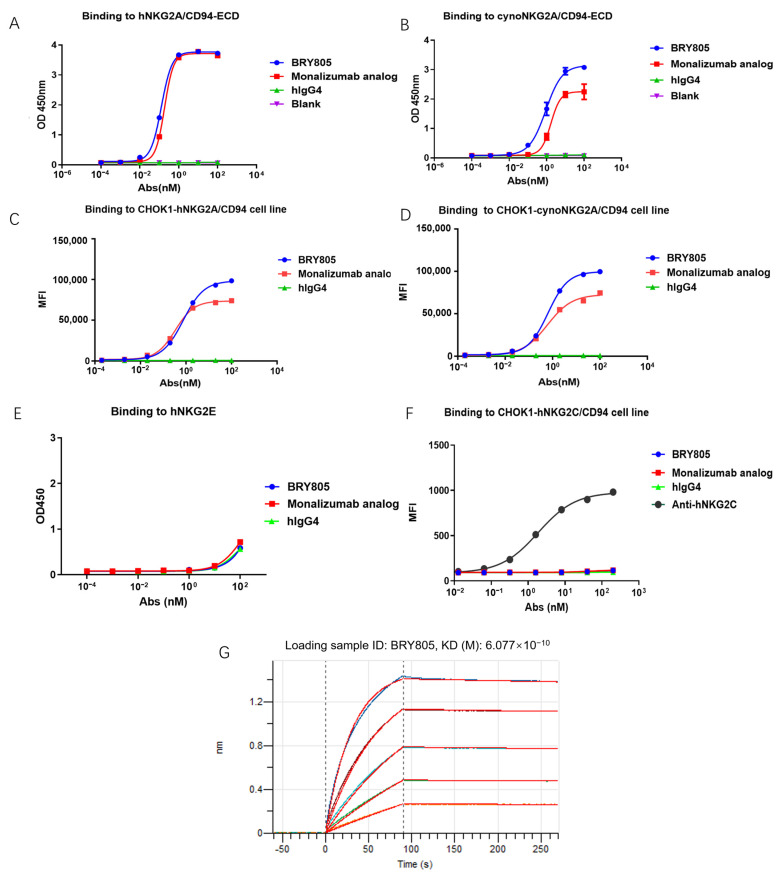
Binding activity of BRY805 to proteins and cells was assessed through various methodologies. (**A**) The interaction of BRY805 with human NKG2A/CD94 extracellular domain (ECD) was quantified using enzyme-linked immunosorbent assay (ELISA). (**B**) The binding of BRY805 to cynomolgus NKG2A/CD94 ECD was evaluated via ELISA. (**C**) Flow cytometry analysis demonstrated the binding of BRY805 to CHOK1 cells overexpressing human NKG2A/CD94. (**D**) The binding of BRY805 to CHOK1 cells overexpressing cynomolgus NKG2A/CD94 was also analyzed using flow cytometry. (**E**) ELISA was employed to determine the binding affinity of BRY805 to human NKG2E. (**F**) Flow cytometry analysis further revealed the binding of BRY805 to CHOK1 cells overexpressing human NKG2C/CD94. (**G**) Analysis of the binding kinetics of BRY805 to the extracellular domain of hNKG2A/CD94 was conducted using Bio-Layer Interferometry.

**Figure 2:**
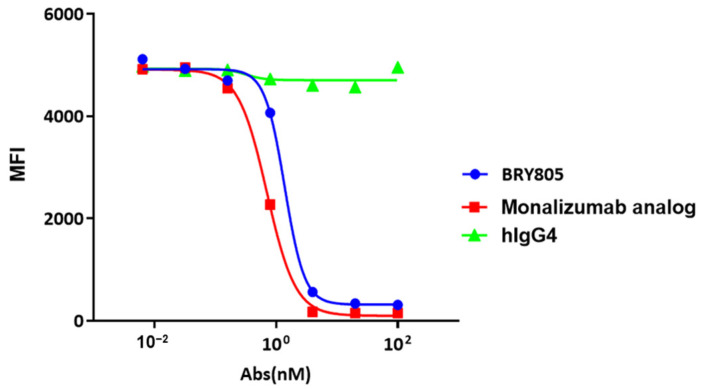
A competitive fluorescence-activated cell-sorting (FACS) experiment utilizing sHLA-E tetramers demonstrated that both BRY805 and monalizumab analog selectively blocked the binding of HLA-E to CD94/NKG2A expressing cells.

Antibodies targeting human NKG2A were developed through the immunization of mice using recombinant NKG2A extracellular domain (ECD) proteins, cells, and DNA. Following screening via NKG2A and NKG2C binding and blocking assays, a positive clone, designated as mAb043, was identified. This monoclonal antibody (mAb) was subsequently humanized by grafting its complementarity-determining regions (CDRs) into human germline frameworks and engineered with a human IgG4 backbone. To enhance antibody stability, prevent the formation of half-antibodies, and eliminate Fc-FcγR interactions, specific amino acid substitutions—S228P, L235E, D265A, and R409K—were introduced. The humanized variant of mAb043, designated as BRY805, was engineered to address the challenge of developing antibodies with selective binding and blocking capabilities for NKG2A, given the 95% sequence identity between human NKG2C and NKG2A, which differ by only a few amino acids in their extracellular domains. Unlike the majority of antibodies screened, BRY805 demonstrates specificity for human NKG2A. It binds to cells expressing human NKG2A and the human NKG2A protein (Figure 1A,C) without interacting with cells expressing human NKG2C or the NKG2E protein (Figure 1E,F). Additionally, BRY805 exhibits binding affinity for cynomolgus NKG2A (Figure 1B,D). BRY805 exhibited a strong affinity for the human NKG2A/CD94 receptor, characterized by an affinity constant (KD) of 0.6 nM (Figure 1G). Subsequently, we evaluated the ability of BRY805 to inhibit the binding of human HLA-E to cells overexpressing human NKG2A. The results demonstrated that BRY805 effectively obstructed the interaction between HLA-E and NKG2A in a dose-dependent manner (Figure 2).

### 3.2. BRY805 Enhances NK92 Cell-Mediated Cytotoxicity

**Figure 3:**
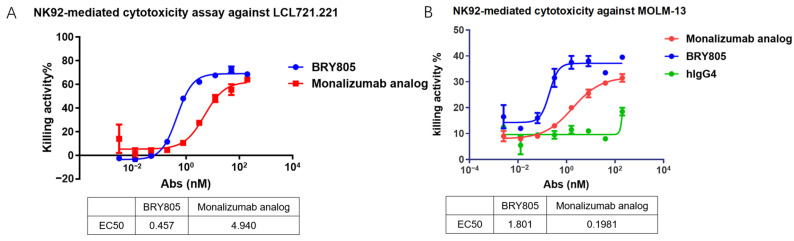
BRY805 enhances the cytotoxicity of NK92 cells against tumor cells. Specifically, (**A**) the B-lymphoblastoid cell line LCL721.221 and (**B**) the myeloid leukemia cell line MOLM-13 were co-incubated with NK92 cells in the presence of serial dilutions of BRY805. The resulting cytotoxic effects were quantified using a time-resolved fluorometer to measure fluorescence.

This study assessed the potential of BRY805 to enhance the cytotoxicity of NK92 cells against cancer cells. Both LCL721.221 B-lymphoblastoid cells and MOLM-13 myeloid leukemia cells are capable of expressing HLA-E, while NK92 cells exhibit high expression levels of NKG2A/CD94. Upon incubation with these cancer cells, NK92 cells demonstrated minimal cytotoxic activity, attributed to the interaction between HLA-E and NKG2A/CD94, which subsequently inhibited NK92 cell cytotoxicity. However, the introduction of BRY805 disrupted the binding between HLA-E and NKG2A/CD94, thereby enabling NK92 cells to effectively target and kill the cancer cells. Notably, BRY805 demonstrated superior potential in enhancing NK92 cell-mediated cytotoxicity compared to a monalizumab analog, exhibiting a tenfold lower EC50 (Figure 3).

### 3.3. BRY805 Enhances Primary NK Cell-Mediated Cytotoxicity

**Figure 4:**
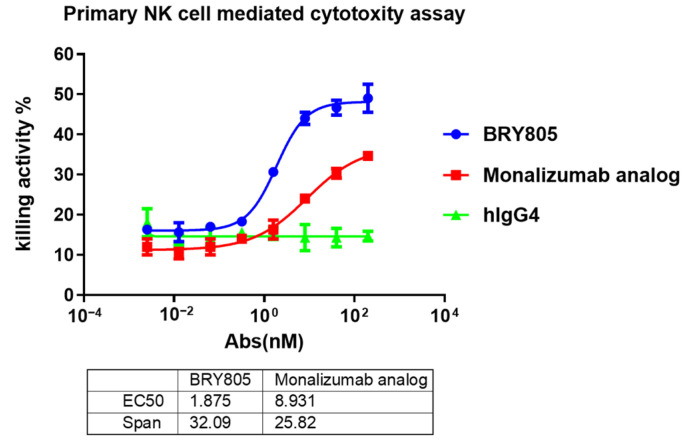
BRY805 augments the cytotoxic activity of primary natural killer (NK) cells against tumor cells. In this study, LCL721.221 cells were co-incubated with primary NK cells in the presence of serial dilutions of BRY805. The cytotoxic effects were quantitatively assessed using a time-resolved fluorometric assay to measure fluorescence.

We then assessed the potential of BRY805 to augment the effector functions of primary natural killer (NK) cells. Primary NK cells were isolated from human peripheral blood mononuclear cells (PBMCs) and screened to obtain NK cells with optimal effector capabilities. LCL721.221 cells were employed as target cells and co-cultured with NK cells at an effector-to-target (E:T) ratio of 2.5:1 in the presence of increasing concentrations of BRY805. Notably, BRY805 induced a concentration-dependent enhancement of NK cell effector functions, as evidenced by increased cytotoxicity against cancer cells. BRY805 demonstrated superior cytotoxic activity compared to a monalizumab analog, with an EC50 of 1.875 nM, which is approximately five times more effective than the monalizumab analog (Figure 4).

### 3.4. BRY805 Synergizes with PD-L1 MAb to Enhance NK Cell-Mediated Cytotoxicity

**Figure 5:**
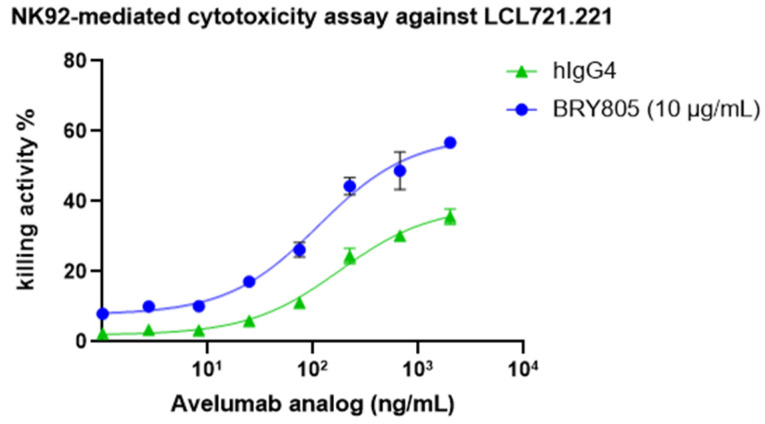
The combination of BRY805 and PD-L1 monoclonal antibody (MAb) significantly enhances the cytotoxic activity of NK92 cells against tumor cells. LCL721.221 cells were co-cultured with NK92 cells in the presence of BRY805 and serially diluted avelumab. Cytotoxicity was quantified by measuring fluorescence using a Tecan microplate reader. BRY805 effectively suppresses tumor growth in a Xenograft tumor model.

To assess the antitumor efficacy of BRY805 in conjunction with a PD-L1 inhibitor, LCL721.221 cells were utilized as target cells and co-cultured with NK92 cells at an effector-to-target (E:T) ratio of 2:1. This was conducted in the presence of BRY805 and varying concentrations of avelumab, alongside control antibodies. The viability of LCL721.221 cells was subsequently determined using Calcein fluorescence signaling. The combination of BRY805 and avelumab demonstrated a statistically significant enhancement in NK cell-mediated cytotoxicity compared to the control antibody (hIgG4) (see Figure 5). The combination of PD-L1 MAb and BRY805 may exert a synergistic effect on the immune response against cancer cells. 

**Figure 6:**
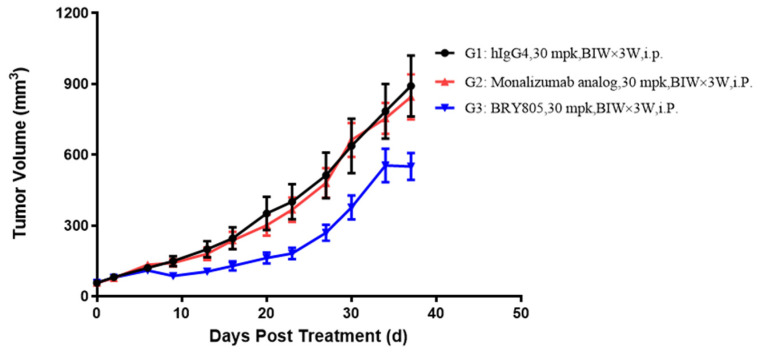
In vivo efficacy of BRY805 evaluated in huHSC-NCG-hIL-5 mice bearing NCI-H1975 tumor xenografts. HuHSC-NCG-hIL-5 mice were subcutaneously injected with 2 × 10^6^ NCI-H1975 tumor cells. Subsequently, molar equivalent doses of the specified antibodies were administered biweekly, and tumor size was measured biweekly.

To evaluate the in vivo efficacy of BRY805, NCI-H1975 cells expressing HLA-E were subcutaneously injected into the flanks of huHSC-NCG-hIL15 mice. These mice are capable of supporting the reconstitution of human natural killer (NK) and T cells through the implantation of human hematopoietic stem cells (HSCs), resulting in a higher NK cell level compared to peripheral blood mononuclear cells (PBMCs) and providing a closer approximation to human biology. However, a limitation of this model is that more than one human HSC is required to reconstitute a sufficient number of mice in a single experiment, which may lead to variability in immune levels. Mice were administered treatments with either a human IgG4 isotype control, a monalizumab analog, or BRY805. As indicated in Table S1, on day 37, the mean tumor volume (mTV) for the isotype control group was 891.56 mm^3^. In comparison, the mTVs for the BRY805 and monalizumab analog groups were 550.38 mm^3^ and 845.04 mm^3^, respectively, with tumor growth inhibitions (TGIs) of 36.04% (*p* < 0.05) and 4.2% (*p* = 0.777). The administration of BRY805 resulted in a statistically significant reduction in tumor growth compared to both the human IgG4 isotype control and the monalizumab analog (Figure 6). Based on the tumor weight measurements on day 37 (refer to Table S2 and Figure S1), BRY805 administered at a dosage of 30 mg/kg demonstrated a statistically significant inhibitory effect on tumor weight increase, with a tumor growth inhibition rate of 36.64% (*p* = 0.014), in comparison to the negative control hIgG4 isotype. In contrast, the monalizumab analog at the same dosage did not exhibit a significant effect, with a tumor growth inhibition rate of −3.84%. Additionally, flow cytometry analysis of human immune cells from the peripheral blood of the mice revealed that the frequency of human CD16+ and CD8+ cells was elevated in mice treated with BRY805 compared to those receiving the hIgG4 isotype control (see Figure S3). These findings indicate that BRY805 demonstrates superior antitumor efficacy in vivo relative to the monalizumab analog. During the course of this experiment, no mortality was observed among the mice, and the rate of change in body weight did not differ significantly across the various treatment groups at the conclusion of this study (refer to Table S3 and Figure S2). This indicates that the administered drugs did not induce a significant reduction in body weight, suggesting that they were well tolerated by the mice.

### 3.5. BRY805 Demonstrates a Favorable Safety Profile

The toxicological profile of BRY805 was evaluated in cynomolgus monkeys. BRY805 exhibited binding affinity to cynomolgus monkey NKG2A with an EC50 of 0.64 nM, which is comparable to its binding affinity to human NKG2A (Figure 1C,D). A Good Laboratory Practice (GLP)-compliant toxicology study was conducted over a 5-week period with repeated dosing, followed by a 6-week recovery phase, in cynomolgus monkeys. The administration of BRY805 was well tolerated across five doses, with no observed abnormal clinical signs or BRY805-related alterations in body weight or food consumption throughout this study. The ophthalmologic examinations and cardiovascular safety pharmacology evaluations revealed no alterations attributable to BRY805. Similarly, hematology and clinical chemistry parameters remained unaffected by BRY805. Furthermore, no BRY805-related effects were observed in gross pathology, relative organ weights, or histopathological assessments. These findings suggest that BRY805 may possess a favorable safety profile.

## 4. Discussion

Immunotherapies have significantly advanced the treatment of cancer patients; however, their effectiveness varies according to tumor indications [14]. The therapeutic strategy of blocking PD-1/PD-L1 results in objective responses in only 15–30% of patients, indicating the presence of additional resistance mechanisms [15]. While the majority of current immunomodulatory strategies have concentrated on augmenting T-cell responses, the distinct ability of NK cells to identify and eliminate tumor cells highlights their substantial potential in cancer treatment. NK cells in cancer are engineered to trigger a complex immune response that ultimately results in protective and enduring immunity against tumors involving various cell types, including T cells [9,16,17]. The HLA-E/NKG2A immune checkpoint axis holds potential as a next-generation immunotherapeutic strategy for cancer [18,19]. The expression of HLA-E, along with the increased levels of CD94/NKG2A observed in tumor-infiltrating lymphocytes (TILs), has been associated with tumor progression, metastasis, and decreased patient survival rates in certain cancers [20,21,22].

Strategies for NKG2A blockade have been developed and validated, particularly in conjunction with anti-PD-L1 antibodies, to improve clinical outcomes in patients with unresectable non-small cell lung cancer [12]. The blockade of NKG2A enhances the antitumor activities of both T cells and NK cells. Additionally, it inhibits the NKG2A ligand, HLA-E, which is overexpressed in the human tumor microenvironment (TME) and contributes to the reduction in lymphocyte expression within the TME [1]. Monalizumab is the first and most extensively studied anti-NKG2A antibody, owing to its demonstrated anticancer activity in early clinical trials. Monalizumab has been shown to enhance the effectiveness of anti-PD-1/PD-L1 inhibition in combination therapy [20]. Research has indicated that monalizumab targets various crucial elements of the immune response [23]. However, the published data [14] suggest that the effectiveness of monalizumab was limited, highlighting the need for the development of a more potent antibody.

In this study, we introduce a novel humanized anti-NKG2A antibody, BRY805. This antibody exhibits high-affinity binding to human NKG2A and effectively disrupts the interaction between NKG2A and HLA-E. Experimental results demonstrate that BRY805 significantly enhances the cytotoxic activity of NK92 or primary NK cells and inhibits tumor growth in vivo, surpassing the efficacy of monalizumab. Additionally, BRY805 has the potential to synergize with anti-PD-1/PD-L1 inhibitors, cetuximab, or trastuzumab in combination therapy.

NKG2C, specific for HLA-E, is an activating receptor of NK cells. HLA-E plays a distinct and pivotal role in modulating the immune response by interacting with either the activating CD94/NKG2C receptor or the inhibitory CD94/NKG2A receptor [24]. Although human inhibitory NKG2A and activating NKG2C receptors share over 95% sequence identity [25], the inhibitory CD94/NKG2A receptor binds to HLA-E with approximately six times greater affinity than CD94/NKG2C. The NKG2A inhibitory receptor plays a pivotal role in immune suppression, suggesting that blocking NKG2A may be a more effective strategy for enhancing immune responses compared to using NKG2C agonists [25,26]. Through an exclusive panning strategy employing recombinant NKG2A and NKG2C proteins, we successfully isolated and characterized human NKG2A inhibitors. Notably, BRY805 specifically binds to NKG2A without interacting with NKG2C. Furthermore, the NKG2C and NKG2E genes exhibit a high degree of genomic similarity (92.1%) [25]; yet, BRY805 does not recognize NKG2E. Given that both NKG2C and NKG2A can form heterodimers with CD94, it is significant that BRY805 does not bind to CD94.

BRY805 demonstrated superior bioactivity in augmenting NK cell-mediated cytotoxicity compared to monalizumab. Notably, for primary NK cells, BRY805 exhibited significantly higher maximum cytotoxicity and a more favorable EC50 than monalizumab. One potential explanation for this observation is that over 90% of NK92 cells express the NKG2A receptor, in contrast to less than 50% of primary NK cells, which not only exhibit lower expression levels of this receptor but are also frequently characterized by dysfunction [27]. Additionally, epitope binning analysis indicates that BRY805 interacts with a distinct epitope compared to monalizumab (Table S4). The in vitro functional assay data for monalizumab were consistent with previously published findings. BRY805 demonstrates significantly superior antitumor efficacy compared to monalizumab, indicating its potential for enhanced clinical application. Conducting in vivo studies targeting NKG2A necessitates a substantial reconstruction of NK cells; otherwise, the quantity of NKG2A+ NK cells will be insufficient for experimental purposes. Although huHSC-NCG-hIL15 mice can achieve a higher level of NK cell reconstitution compared to PBMC mice, this level remains lower than that observed in human peripheral blood. Given the limited in vitro efficacy of monalizumab, it fails to produce a notable effect in vivo. Attempts to utilize an NKG2A humanized mouse model were unsuccessful, as NKG2A+ NK cells were barely detectable, and no efficacy was observed. Furthermore, it has been documented that peptides presented by HLA-E modulated its interaction with the NKG2A/CD94 receptor complex. Monalizumab exhibited diminished effectiveness in augmenting natural killer (NK) cell-mediated cytotoxicity against target cells presenting HLA-G peptides on HLA-E, in contrast to cells expressing HLA-E in association with peptides from HLA-A, HLA-B, and HLA-C [13]. This finding implies that BRY805 may offer a therapeutic advantage for patients who exhibit resistance to immune checkpoint inhibitor (ICI) therapies by specifically targeting NKG2A. Further investigations are underway to elucidate the impact of BRY805 on CD8+ T cell cytotoxic function. In peripheral blood mononuclear cells (PBMCs) from healthy donors, the NKG2A+ population constituted less than 2% of CD3 + CD8+ T cells. In contrast, the expression level of NKG2A was significantly elevated in T cells isolated from peripheral blood mononuclear cell (PBMC) samples of cancer patients, reaching levels as high as 16%.

Toxicological studies suggest that BRY805 possesses a favorable safety profile; however, it may also present risks associated with immune-related adverse events (irAEs), such as hyperactivation of immune cells, off-target effects, increased susceptibility to infections, and hematologic effects akin to other immune checkpoint inhibitors. Therefore, vigilant monitoring and timely management of these potential side effects are essential during NKG2A-targeted therapies. Collectively, the data presented here demonstrate that BRY805 can activate natural killer (NK) cells, thereby enhancing NK cell-mediated cytotoxicity against HLA-E+ tumor cells, synergizing with PD-L1 inhibitors, cetuximab or trastuzumab, and inhibiting tumor growth in vivo. Additionally, toxicological assessments in non-human primates (NHP) indicate that BRY805 possesses a favorable safety profile. These findings provide a rationale for the continued development of BRY805 in clinical antitumor therapy. By preventing NKG2A-mediated inhibition, patients could experience a more robust immune response against tumors, particularly those that express HLA-E to evade immune detection. Currently, BRY805 is being evaluated in a Phase 1 clinical trial in China.

## 5. Conclusions

BRY805 is a novel anti-NKG2A antibody that specifically targets the NKG2A/CD94 receptor, effectively blocking the interaction between HLA-E and NKG2A/CD94. This antibody has the capacity to activate natural killer (NK) cells and works synergistically with PD-L1 inhibitors, cetuximab or trastuzumab, to augment NK cell-mediated cytotoxicity against tumor cells. In both in vitro and in vivo studies, BRY805 demonstrates superior efficacy in cancer immunotherapy compared to monalizumab. BRY805 demonstrates substantial potential for cancer patients, especially when used in conjunction with other therapies, such as checkpoint inhibitors and EGFR-targeting agents, to address resistance to existing treatments. Its potential applications span a variety of cancers, including lung, head and neck, colorectal, and hematological malignancies. Furthermore, its favorable safety profile positions BRY805 as a promising novel strategy in the field of cancer immunotherapy.

## Data Availability

The original contributions presented in this study are included in the supplementary material; further inquiries can be directed to the corresponding author.

## Supplementary Materials

The following supporting information can be downloaded at https://www.mdpi.com/article/10.3390/antib13040093/s1, Figure S1: Tumor weights of NCI-H1975 xenograft models in huHSC-NCG-IL15 mice treated with BRY805 or a monalizumab analog were assessed on day 37; Figure S2: Body weight change in NCI-H1975 xenograft huHSC-NCG-ILl5 mice treated with BRY805 or monalizumab analog; Figure S3: Human hematopoiesis was investigated in NCI-H1975 xenograft huHSC-NCG-IL15 mice. The development of human leukocytes was assessed through peripheral blood analysis via flow cytometry at the conclusion of this experiment; Table S1: The efficacy results of tumor volume at day 37; Table S2: The efficacy results of tumor weight at day 37; Table S3: The tolerance of mice at day 37; Table S4: Epitope binning of BRY805; Table S5: Research data for Figure 4; Table S6: Research data for Figure 5; Table S7: Research data for Figure 6.

## References

[1] Dutta S., Ganguly A., Chatterjee K., Spada S., Mukherjee S. (2023). Targets of Immune Escape Mechanisms in Cancer: Basis for Development and Evolution of Cancer Immune Checkpoint Inhibitors. Biology.

[2] Mingari M.C., Pietra G., Moretta L. (2019). Immune Checkpoint Inhibitors: Anti-NKG2A Antibodies on Board. Trends Immunol..

[3] André P., Denis C., Soulas C., Bourbon-Caillet C., Lopez J., Arnoux T., Bléry M., Bonnafous C., Gauthier L., Morel A. (2018). Anti-NKG2A mAb Is a Checkpoint Inhibitor That Promotes Anti-Tumor Immunity by Unleashing Both T and NK Cells. Cell.

[4] Yang Y., Liu Z., Wang H., Zhang G. (2021). HLA-E Binding Peptide as a Potential Therapeutic Candidate for High-Risk Multiple Myeloma. Front. Oncol..

[5] Liu X., Song J., Zhang H., Liu X., Zuo F., Zhao Y., Zhao Y., Yin X., Guo X., Wu X. (2023). Immune Checkpoint HLA-E:CD94-NKG2A Mediates Evasion of Circulating Tumor Cells from NK Cell Surveillance. Cancer Cell.

[6] De Kruijf E.M., Sajet A., Van Nes J.G.H., Natanov R., Putter H., Smit V.T.H.B.M., Liefers G.J., Van Den Elsen P.J., Van De Velde C.J.H., Kuppen P.J.K. (2010). HLA-E and HLA-G Expression in Classical HLA Class I-Negative Tumors Is of Prognostic Value for Clinical Outcome of Early Breast Cancer Patients. J. Immunol..

[7] Levy E.M., Bianchini M., Von Euw E.M., Barrio M.M., Bravo A.I., Furman D., Domenichini E., Macagno C., Pinsky V., Zucchini C. (2008). Human Leukocyte Antigen-E Protein Is Overexpressed in Primary Human Colorectal Cancer. Int. J. Oncol..

[8] Chen Y., Xin Z., Huang L., Zhao L., Wang S., Cheng J., Wu P., Chai Y. (2020). CD8+ T Cells Form the Predominant Subset of NKG2A+ Cells in Human Lung Cancer. Front. Immunol..

[9] Kamiya T., Seow S.V., Wong D., Robinson M., Campana D. (2019). Blocking Expression of Inhibitory Receptor NKG2A Overcomes Tumor Resistance to NK Cells. J. Clin. Investig..

[10] Van Hall T., André P., Horowitz A., Ruan D.F., Borst L., Zerbib R., Narni-Mancinelli E., Van Der Burg S.H., Vivier E. (2019). Monalizumab: Inhibiting the Novel Immune Checkpoint NKG2A. J. Immunother. Cancer.

[11] Galot R., Le Tourneau C., Saada-Bouzid E., Daste A., Even C., Debruyne P., Henry S., Zanetta S., Rutten A., Licitra L. (2021). A Phase II Study of Monalizumab in Patients with Recurrent/Metastatic Squamous Cell Carcinoma of the Head and Neck: The I1 Cohort of the EORTC-HNCG-1559 UPSTREAM Trial. Eur. J. Cancer.

[12] Herbst R.S., Majem M., Barlesi F., Carcereny E., Chu Q., Monnet I., Sanchez-Hernandez A., Dakhil S., Camidge D.R., Winzer L. (2022). COAST: An Open-Label, Phase II, Multidrug Platform Study of Durvalumab Alone or in Combination With Oleclumab or Monalizumab in Patients With Unresectable, Stage III Non–Small-Cell Lung Cancer. J. Clin. Oncol..

[13] Battin C., Kaufmann G., Leitner J., Tobias J., Wiedermann U., Rölle A., Meyer M., Momburg F., Steinberger P. (2022). NKG2A -checkpoint Inhibition and Its Blockade Critically Depends on Peptides Presented by Its Ligand HLA-E. Immunology.

[14] Thommen D.S., Schumacher T.N. (2018). T Cell Dysfunction in Cancer. Cancer Cell.

[15] Patel S.P., Othus M., Chae Y.K., Giles F.J., Hansel D.E., Singh P.P., Fontaine A., Shah M.H., Kasi A., Baghdadi T.A. (2020). A Phase II Basket Trial of Dual Anti–CTLA-4 and Anti–PD-1 Blockade in Rare Tumors (DART SWOG 1609) in Patients with Nonpancreatic Neuroendocrine Tumors. Clin. Cancer Res..

[16] Choucair K., Duff J.R., Cassidy C.S., Albrethsen M.T., Kelso J.D., Lenhard A., Staats H., Patel R., Brunicardi F.C., Dworkin L. (2019). Natural Killer Cells: A Review of Biology, Therapeutic Potential and Challenges in Treatment of Solid Tumors. Future Oncol..

[17] Shimasaki N., Coustan-Smith E., Kamiya T., Campana D. (2016). Expanded and Armed Natural Killer Cells for Cancer Treatment. Cytotherapy.

[18] Borst L., van der Burg S.H., van Hall T. (2020). The NKG2A–HLA-E Axis as a Novel Checkpoint in the Tumor Microenvironment. Clin. Cancer Res..

[19] Fisher J., Doyle A., Graham L., Khakoo S., Blunt M. (2022). Disruption of the NKG2A:HLA-E Immune Checkpoint Axis to Enhance NK Cell Activation against Cancer. Vaccines.

[20] Abd Hamid M., Wang R.-Z., Yao X., Fan P., Li X., Chang X.-M., Feng Y., Jones S., Maldonado-Perez D., Waugh C. (2019). Enriched HLA-E and CD94/NKG2A Interaction Limits Antitumor CD8+ Tumor-Infiltrating T Lymphocyte Responses. Cancer Immunol. Res..

[21] Sun C., Xu J., Huang Q., Huang M., Wen H., Zhang C., Wang J., Song J., Zheng M., Sun H. (2017). High NKG2A Expression Contributes to NK Cell Exhaustion and Predicts a Poor Prognosis of Patients with Liver Cancer. OncoImmunology.

[22] Seliger B., Jasinski-Bergner S., Quandt D., Stoehr C., Bukur J., Wach S., Legal W., Taubert H., Wullich B., Hartmann A. (2016). HLA-E Expression and Its Clinical Relevance in Human Renal Cell Carcinoma. Oncotarget.

[23] Salomé B., Sfakianos J.P., Ranti D., Daza J., Bieber C., Charap A., Hammer C., Banchereau R., Farkas A.M., Ruan D.F. (2022). NKG2A and HLA-E Define an Alternative Immune Checkpoint Axis in Bladder Cancer. Cancer Cell.

[24] Lauterbach N., Wieten L., Popeijus H., Voorter C., Tilanus M.G.J. (2015). HLA-E Regulates the NKG2C+ Natural Killer Cell Function through Presentation of a Restricted Peptide Repertoire. Hum. Immunol..

[25] Siemaszko J., Marzec-Przyszlak A., Bogunia-Kubik K. (2023). Activating NKG2C Receptor: Functional Characteristics and Current Strategies in Clinical Applications. Arch. Immunol. Ther. Exp..

[26] Cauli A., Dessole G., Piga M., Angioni M.M., Pinna S., Floris A., Congia M., Mascia E., Paladini F., Tedeschi V. (2018). Expression Analysis of HLA-E and NKG2A and NKG2C Receptors Points at a Role for Natural Killer Function in Ankylosing Spondylitis. RMD Open.

[27] Fabian K.P., Hodge J.W. (2021). The Emerging Role of Off-the-Shelf Engineered Natural Killer Cells in Targeted Cancer Immunotherapy. Mol. Ther.-Oncolytics.

