# Supplementary material for: Generation, Characterization, and Preclinical Studies of a Novel NKG2A-Targeted Antibody BRY805 for Cancer Immunotherapy
Source: Antibodies (Basel). 2024 Nov 20;13(4):93. doi: 10.3390/antib13040093 (PMC11587108; doi:10.3390/antib13040093)
Supplement: Supplementary file 1 [file antibodies-13-00093-s001.zip › antibodies-3195274-supplementary.pdf]

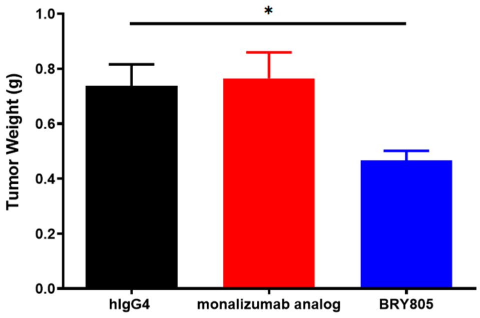

Figure S1. Tumor weights of NCI-H1975 xenograft models in huHSC-NCG-IL15 mice treated with BRY805 or a monalizumab analog were assessed on day 37.

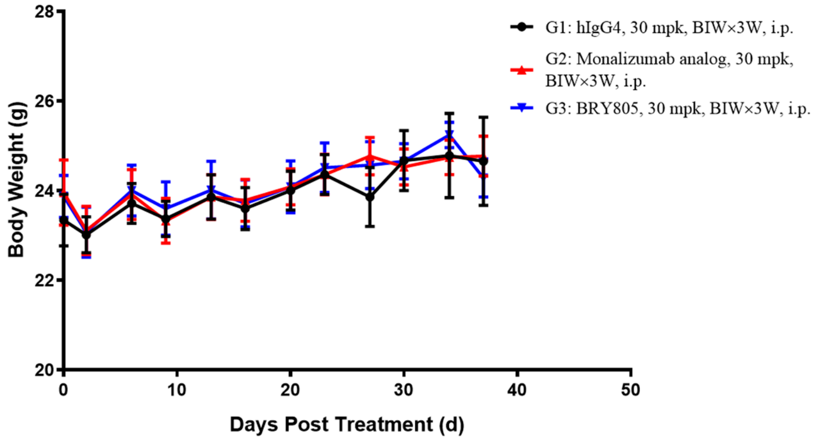

Figure S2. Body weight change of NCI-H1975 xenograft huHSC-NCG-ILl5 mice treated with BRY805 or monalizumab analog

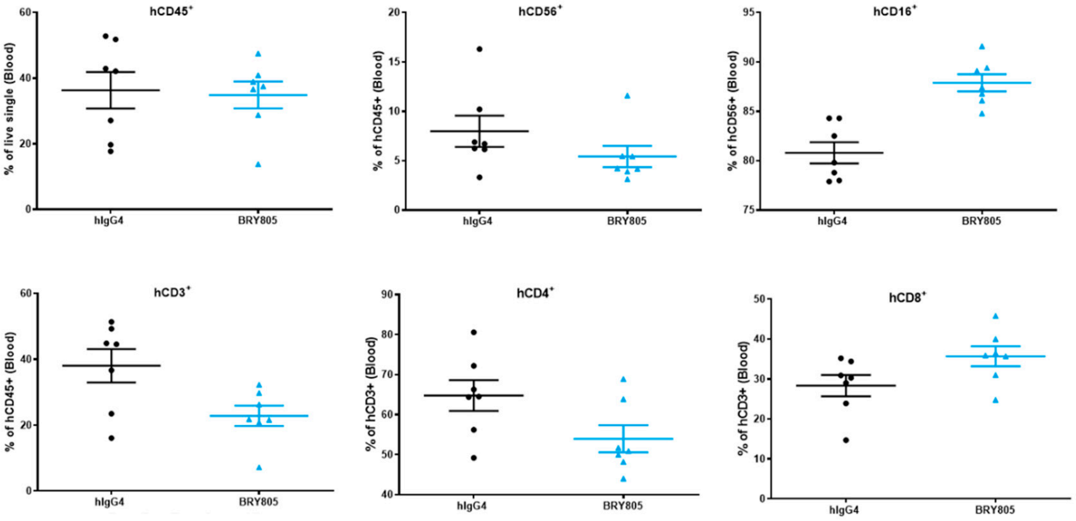

Figure S3. Human hematopoiesis was investigated in NCI-H1975 xenograft huHSC-NCG-IL15 mice. The development of human leukocytes was assessed through peripheral blood analysis via flow cytometry at the conclusion of the experiment.

Table S1. The efficacy results of tumor volume at day 37

Group	Drugs	Administration (mg/kg)	TV (mean ± SEM) mm^3	TGI_{TV}	pValue
G1	hIgG4	30	891.56 ±129.04	-	-
G2	monalizumab analog	30	845.044±95.59	4.2%	0.777
G3	BRY805	30	550.38±56.55	36.04%	0.041*

Table S2. The efficacy results of tumor weight at day 37

Group	Drugs	tumor weight (mean ± SEM)g	TGI_{TW}	pValue
G1	hIgG4	0.74±0.08	-	-
G2	monalizumab analog	0.77±0.09	-3.84%	0.822
G3	BRY805	0 .47±0.03	36.64%	0.014*

Table S3. The tolerance of mice at day 37

Group	Drugs	Number of mice Day0/Day37	Day 0 Body Weight (mean ± SEM) g	Day 37 Body Weight (mean ± SEM) g	Body Weight Change (mean)
G1	hIgG4	7/7	23.34 ± 0.58	24.66 ± 0.98	5.73%
G2	monalizumab analog	7/7	23.96 ± 0.73	24.77 ± 0.45	3.70%
G3	BRY805	7/7	23.87 ± 0.47	24.29 ± 0.43	1.86%

Table S4. Epitope binning of BRY805

Ab	BRY805	monalizumab analog
BRY805	94%	96%
monalizumab analog	20%	77%

Research data is presented as follows:

Table S5: Research data for Fig. 4

	Abs (nM)	1	2	3	4	5	6	7	8	9	10	11	12
A	200	hIgG4			BRY805			monalizumab analog			NK+LCL		
B	40												
C	8												
D	1.6										NK+LCL+Lysis		
E	0.32										NK+LCL		
F	0.064										LCL+Lysis		
G	0.0128										LCL only		
H	0.00256										buffer		

	Abs (nM)	1	2	3	4	5	6	7	8	9	10	11	12
A	200	338057	346161	337450	423774	421861	399899	387268	380821	385026	328857	331645	326925
B	40	331020	346884	340853	414113	412784	401955	375007	378244	368429	327872	335768	331251
C	8	326614	342317	350262	402332	409801	399546	365272	358933	359192	332205	336615	323350
D	1.6	340602	336710	347377	378400	373011	372790	351262	335512	345373	512242	555876	533656
E	0.32	341730	342531	342199	348539	346997	349199	340272	335841	337935	341508	336689	333303
F	0.064	348902	332449	337084	343639	346938	344663	342938	329686	329530	524676	522007	528941
G	0.0128	336863	335310	344217	341337	351074	333523	335680	328917	328805	307190	310533	307798
H	0.00256	362889	338866	341742	345114	344755	341539	339918	338200	326742	97290	96375	96293

	Abs (nM)	1	2	3	4	5	6	7	8	9	10	11	12
A	200	14%	17%	13%	53%	52%	42%	36%	33%	35%	9%	11%	8%
B	40	10%	18%	15%	49%	48%	43%	31%	32%	28%	9%	13%	10%
C	8	8%	16%	19%	43%	47%	42%	26%	23%	23%	11%	13%	7%
D	1.6	15%	13%	18%	32%	30%	30%	20%	12%	17%			
E	0.32	15%	16%	16%	18%	18%	19%	15%	13%	14%	15%	13%	11%
F	0.064	19%	11%	13%	16%	18%	17%	16%	10%	10%			
G	0.0128	13%	12%	16%	15%	20%	12%	13%	9%	9%			
H	0.00256	25%	14%	15%	17%	17%	15%	14%	14%	8%			

Table S6: Research data for Fig.5

Avelumab analog (ng/mL)	Control mAb (IgG4)			BRY805 (10 μg/mL)		
0	2.420792	2.411497	2.172129	7.528857	9.736619	6.915331
2.743484	4.953909	2.969247	2.332482	11.71198	9.504223	9.041755
8.230453	2.736851	3.829112	3.427067	11.07522	10.78937	8.669921
24.69136	7.417307	4.932993	5.632505	18.75126	17.19421	15.64645
74.07407	11.86072	11.956	9.727323	27.82632	27.04083	23.90813
222.2222	26.69455	24.32412	22.27438	47.09892	42.31157	43.74777
666.6667	30.74522	30.60346	29.31831	50.90092	52.72291	42.72291
2000	38.20745	34.89581	33.89651	57.824	55.36293	57.05709

Table S7: Research data for Fig.6

ys	G1:IgG4,30 mpk,BIW×3W,i.p.							G2: Monalizumab analog,30 mpk,BIW×3W,i.P.							G3:BRY805,30 mpk,BIW×3W,i.P.						
0	45.46	50.34	56.22	58.84	61.37	64.27	66.76	49	50.27	53.8	57.52	60.62	64.11	67.91	43.01	50.96	52.17	58.24	63.21	66.36	68.54
2	65.76	67.76	83.38	90.26	83.01	87.41	94.94	84.58	72.17	64.22	76.21	80.67	74.37	92.06	56.62	90.57	74.3	79.17	81.25	105.8	69.39
6	81.75	78.62	120.79	148.65	103.11	144.39	169.6	145.16	154.9	107.97	125.75	134.58	112.05	160.33	100.21	95.43	124.82	105.37	141.58	113.11	92.87
9	81.14	76.56	165.97	199.59	130.66	174.32	220.96	144.39	188.73	81.33	104.31	128.74	154.2	190.14	85.06	65.89	103.54	72.62	133.61	93.66	52.5
13	80.43	93.86	278.34	271.79	136.96	261.11	276.2	191.71	190.23	83.27	113.96	166.21	226.91	291.75	123.72	78.42	138.13	103.75	153.08	81.09	56.05
16	100.88	94.84	373.86	327.59	159.88	310.4	355.58	267.78	280.49	110.83	131.72	193.14	300.5	375.86	165.74	79.61	173.21	117.63	200.64	104.29	65.61
20	135.72	128.56	495.11	530.8	198.49	458.12	512.43	339.54	310.29	148.58	171.09	260.22	445.44	433.67	204.24	87.57	227.91	175.15	222.92	133.73	87.78
23	159.26	163.27	577.73	555.28	259.74	569.54	523.04	432.43	448.46	193.98	189.31	314.03	533.75	461.72	259.64	104.64	245.47	186.07	220.83	147.42	108.27
27	207.05	274.58	850.44	711.67	285.91	704.32	554.76	499.22	524.51	286.66	262.83	435.68	672.06	683.39	360.48	175.2	306.73	329.6	358.05	198.75	157.19
30	277.55	365.98	970.95	918.12	450.06	975.89	502.34	745.75	629.23	416.1	422.29	677.32	854.47	895.61	465.33	181.95	311.25	573.41	484.15	321.62	302.92
34	403.05	528.46	1084.24	1086.84	653.3	1129.95	602.7	856.03	702.56	673.03	551.67	573.4	985.76	940.29	604.21	269.5	638.89	834.94	602.73	572.25	360.58
37	507.14	604.62	1331.99	1239.14	628.15	1157.67	772.24	968.03	854.57	587.48	669.8	579.9	1271.6	983.93	588.18	336.84	644.31	760.54	598.2	553.1	371.45
